# Electrical Brain Responses Reveal Sequential Constraints on Planning during Music Performance

**DOI:** 10.3390/brainsci9020025

**Published:** 2019-01-28

**Authors:** Brian Mathias, William J. Gehring, Caroline Palmer

**Affiliations:** 1 Department of Psychology, McGill University, Montreal, QC H3A 1B1, Canada; 2 Research Group Neural Mechanisms of Human Communication, Max Planck Institute for Human Cognitive and Brain Sciences, 04103 Leipzig, Germany; 3 Department of Psychology, University of Michigan, Ann Arbor, MI 48109, USA; wgehring@umich.edu

**Keywords:** sensorimotor learning, sequence production, sequence planning, feedback monitoring, EEG, N1, FRN, music performance, music cognition, altered auditory feedback

## Abstract

Elements in speech and music unfold sequentially over time. To produce sentences and melodies quickly and accurately, individuals must plan upcoming sequence events, as well as monitor outcomes via auditory feedback. We investigated the neural correlates of sequential planning and monitoring processes by manipulating auditory feedback during music performance. Pianists performed isochronous melodies from memory at an initially cued rate while their electroencephalogram was recorded. Pitch feedback was occasionally altered to match either an immediately upcoming Near-Future pitch (next sequence event) or a more distant Far-Future pitch (two events ahead of the current event). Near-Future, but not Far-Future altered feedback perturbed the timing of pianists’ performances, suggesting greater interference of Near-Future sequential events with current planning processes. Near-Future feedback triggered a greater reduction in auditory sensory suppression (enhanced response) than Far-Future feedback, reflected in the P2 component elicited by the pitch event following the unexpected pitch change. Greater timing perturbations were associated with enhanced cortical sensory processing of the pitch event following the Near-Future altered feedback. Both types of feedback alterations elicited feedback-related negativity (FRN) and P3a potentials and amplified spectral power in the theta frequency range. These findings suggest similar constraints on producers’ sequential planning to those reported in speech production.

## 1. Introduction

Many everyday behaviors, such as having a conversation, writing a note, and driving a car, involve the production of action sequences. A core tenet of theories of sequential behavior is that, in order to produce action sequences quickly and accurately, individuals must plan appropriate movements prior to their execution [1]. Evidence for future-oriented planning during sequential tasks comes from anticipatory ordering errors, in which upcoming sequence events are produced earlier in the sequence than intended. Documented in both speech production [2,3] and music performance [4,5,6], anticipatory errors suggest that producers have access to a range of upcoming events in a sequence at any given time during production. The span of sequence positions between an event’s correct (intended) position and its incorrect (error-produced) position is taken to indicate a producer’s range of planning, due to the items’ simultaneous accessibility [5]. Serial ordering errors during music performance tend to arise more often from closer sequence distances than from farther distances [7,8,9]. This tendency suggests that producers have increased access to events intended for nearer in the future compared to events that are intended for farther ahead in the future [8]. These proximity constraints on planning have been attributed to interference of future events with current events during memory retrieval, decay of item information over time, and individual differences in producers’ working memory spans [8,10,11]. Sequential models of sequence planning in both speech [12] and music [8] use the term “planning gradient” to refer to a decrease in memory activation of upcoming sequence events as the distance from the current event increases.

In addition to planning upcoming units of speech and music during production, speakers and musicians monitor the perceptual outcomes of their previous productions. In order to monitor perceptual outcomes during auditory-motor tasks, producers compare perceived auditory feedback with an intended auditory outcome [10]. Theoretical approaches to feedback monitoring have focused heavily on the concept of internal models, or representations that simulate a response to estimate an outcome (for a review, see [13]). Internal models are thought to arise from interactions between bottom-up, incoming sensory information and top-down expectations or predictions formed by the motor system during production [14]. A framework known as “predictive coding” assumes that the goal of production is to minimize prediction error (i.e., mismatches between predictions that are generated by an internal model and sensory information originating in the environment) [15,16]. Musicians possess strong associations between musical actions and their sensory outcomes [17], which may explain why the perception of inaccurate auditory feedback during the production of auditory-motor sequences can disrupt production [18]. Mismatches between auditory feedback from musician’s planned movements [7] as well as nonmusicians’ planned movements can generate prediction errors, evidenced by an increasing error-related negativity [19]. Experimentally altering the contents of pitch feedback during music performance can disrupt the regular timing of key presses [20] and increase pitch error rates [21,22]. The computer-controlled removal of auditory feedback in laboratory environments does not disrupt well-learned performance; musicians can continue performing well-learned music when auditory feedback is removed [23,24], and altering feedback so that it is highly different from expected feedback has little effect on a previously learned performance [25]. Performance is disrupted when the altered auditory feedback is similar to the planned events [25,26]. Thus, current evidence suggests that disruption caused by altered auditory feedback may depend on similarity-based interference with planned sequence representations, leading to a novel prediction: If the planning of future events occurs in a graded fashion (higher activation for immediately upcoming events compared to more distant future events), then altered feedback that matches immediately upcoming events should disrupt performance more than altered feedback matching sequentially distant events.

To our knowledge, no studies in the domain of speech production have tested neural effects of hearing upcoming linguistic content that is presented sooner than expected while speaking. This may be due to the difficulties of independently manipulating auditory feedback during concurrent speech production. Auditory feedback during speech production can be electronically delayed, so that instead of hearing current feedback, one hears feedback that matches previous utterances. Presenting linguistic content that matches upcoming utterances is more difficult, however, because it requires the presentation of speech content that has not yet been produced by the speaker. In electronic music performance, one can present auditory feedback that matches future keypresses, due to a simpler sound production apparatus. One study examined musicians’ neural responses to future-oriented altered auditory feedback as they performed tone sequences on a piano [7]. Occasional alterations in auditory feedback were presented that matched upcoming (future) events as pianists performed melodies from memory. An example of future-oriented feedback is if a pianist was currently producing tone A and was planning to produce tone B later in the sequence, tone B would be presented auditorily when the pianist’s hand struck the A-key on the keyboard. Future-oriented feedback pitches elicited larger (event-related potential (ERP)) responses than altered feedback that matched previous (past) events, and amplitudes of the ERPs elicited by the altered feedback pitches correlated with the amount of temporal disruption elicited in the pianists’ key presses [7]. It is unknown, however, whether disruptive effects of altered auditory feedback that match future events depend on an individual’s planning gradient: If producers’ plans are biased toward the activation of immediately upcoming events compared to events planned for the distant future, then we would expect pitch feedback that matches immediate future events to generate greater similarity-based interference, and in turn greater performance disruption, than future-oriented feedback that matches distant future events. Thus, future-oriented theories of planning predict greater performance disruption for altered feedback that matches near future events compared to far future events.

Several studies have suggested that sensory processing of altered auditory feedback during production is marked by early- to middle-latency ERP responses to tone onsets [27,28,29]. N1 and P2 ERP components in particular are sensitive to whether speech or tones are generated by oneself versus others [30,31,32]. The N1 is a negative-going ERP component that peaks at about 100 ms following sound onsets and is followed by the positive-going P2 component [33,34]. Amplitudes of these components are more negative when sounds are generated by others than when they are self-generated, which is thought to reflect motor-induced suppression of auditory cortical processing [35,36]. Perceptual studies have demonstrated that N1 and P2 amplitudes also become more negative (larger N1 and smaller P2) in response to tones that are selectively attended to, compared to unattended tones, suggesting a role of these components in early auditory sensory processing [37,38,39,40]. N1 and P2 waves occur in quick succession about 50–150 ms following sound onsets, and arise from several temporally overlapping, spatially-distributed sources, with primary generators in the auditory cortices and planum temporale [33,34,41,42,43]. Thus, N1 and P2 amplitudes may serve as a proxy for the degree to which sensory processing of auditory feedback is suppressed during sound production: A negative-going shift in amplitudes occurs when processing of auditory feedback is enhanced, and a positive-going shift occurs when processing of auditory feedback is suppressed. Combined with the notion that future-oriented feedback that matches near future events may generate greater similarity-based interference than feedback matching far future events, this principle leads to the prediction that altered auditory feedback that matches near future events should decrease the expected N1 and P2 suppression compared to altered feedback that matches far future events.

Additional ERP components linked to action-related expectations are elicited when sensory feedback indicates that an action has resulted in an unexpected outcome. Frontally maximal feedback-related negativities (FRNs) are elicited roughly 150–250 ms following the unexpected outcome in music performance tasks [44,45,46,47], as well as during other tasks, such as reward prediction and monetary gambling tasks [48]. FRN amplitudes may be associated with the degree to which unexpected feedback violates a producer’s feedback-related expectations [49,50,51,52,53]. The FRN component often co-occurs with neural oscillations in the theta frequency range (4–8 Hz), thought to reflect the implementation of cognitive control [54,55,56]. The FRN is typically followed by a frontally-maximal P3a component, which peaks around 300–500 ms following the onset of unexpected feedback. The P3a may reflect the updating of stimulus memory representations [57,58], decision-making processes [59,60], and voluntary shifts of attention to unexpected stimuli [61,62]. If altered auditory feedback during music performance triggers the emergence of a more cognitively-controlled (e.g., deliberative, goal-directed, model-based, prefrontal) state, as opposed to a habitual (e.g., automatic, model-free, striatal) performance state [63], then we would expect theta frequency activity to be enhanced following any feedback alterations, and to be accompanied by FRN and P3a potentials. A benefit of extracting theta band activity related to the FRN is that it can account for potential overlap of neighboring FRN and P3a potentials in the ERP waveform [56,64].

The current study investigated the relationship between performers’ planning and feedback monitoring processes by presenting altered auditory feedback corresponding to upcoming (future) sequence events during music performance. The timing of pianists’ key presses in response to altered auditory feedback pitches was measured. Pianists memorized and performed isochronous melodic sequences on an electronic keyboard while hearing feedback triggered by their key presses over headphones. Altered pitch feedback was manipulated in four conditions: Future +1 (“near future”), future +2 (“far future”), noncontextual, and baseline. In the future +1 condition, participants heard an altered pitch presented at the current location that matched the intended (memorized) pitch at the next location in the sequence. In the future +2 condition, participants heard an altered pitch presented at the current location that matched the intended (memorized) pitch at the location two events ahead of the current location. In the noncontextual condition, participants heard a pitch that was not present in the sequence; this control condition tested effects of hearing an altered feedback pitch that was unrelated to performers’ planning processes. Finally, in the baseline condition, participants heard the expected auditory feedback with no pitch alterations. 

We tested three predictions: First, near future (future +1) altered auditory feedback was expected to induce greater interference with the production of currently planned events than far future (future +2) altered auditory feedback. This prediction is based on producers’ use of planning gradients, in which plans are weighted toward near compared to distant sequence events [8,12]. Pianists were therefore expected to show greater temporal disruption following future +1 altered auditory feedback compared to future +2 altered feedback. Second, we expected performance disruption to be associated with decreased N1 and P2 suppression following future +1 feedback compared to future +2 feedback. Third, future +1, future +2, and noncontextual altered feedback pitches were expected to elicit FRN and P3a ERP components (relative to the baseline condition), as well as corresponding theta oscillations within the timeframe of the FRN. 

## 2. Materials and Methods

### 2.1. Participants

Twenty-eight right-handed adult pianists with at least 6 years of private piano instruction were recruited from the Montreal community. Four participants were excluded from analysis due to insufficient data after trials performed from memory that contained pitch errors (*n* = 3) or EEG artifacts (*n* = 1) were removed. The remaining 24 pianists (15 women, age *M* = 21.1 years, SD = 2.7 years) had between 6 and 20 years of piano lessons (*M* = 11.5 years, SD = 3.9 years). Participants reported having no hearing problems. Two of the pianists reported possessing absolute pitch. Participants provided written informed consent, and the study was reviewed by the McGill University Research Ethics Board.

### 2.2. Stimulus Materials

**Figure 1:**
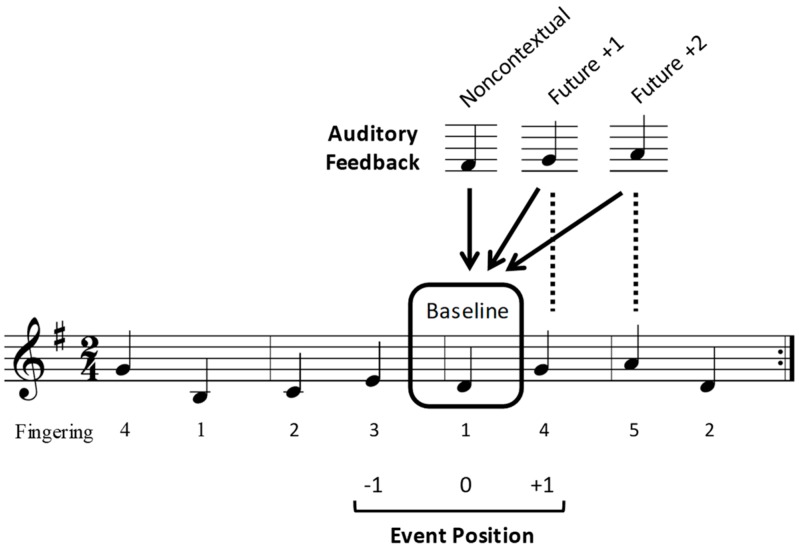
Example of a notated stimulus melody. Sample altered feedback pitches for the four auditory feedback conditions (baseline, noncontextual, future +1, and future +2), and the three target event positions (−1, 0, +1) over which interonset intervals (IOIs) and event-related potentials (ERPs) were analyzed are shown. Target event positions are numbered with respect to the distance of the altered feedback from its intended sequence position. Arrows show the location at which the altered feedback pitches occurred, and dashed lines indicate the origin of the altered feedback pitches.

Four novel melodies that were notated in a binary meter (2/4 time signature), conforming to conventions of Western tonal music, were used in the study. An example of a melody is shown in Figure 1. All melodies were isochronous (containing only 8 quarter notes), were notated for the right hand, and were designed to be repeated without stopping 3 times in each trial (totaling 24 quarter-note events). Each stimulus melody was composed to have no repeating pitches within two sequence positions. The 4 melodies were composed in the keys of G major, D minor, C major, and B minor. Suggested fingering instructions were also notated. 

During the experiment, auditory feedback pitches triggered by participants’ key presses while performing the melodies were occasionally replaced by an altered pitch. The altered pitches were chosen from the same diatonic key as the original melody to maintain the melodic contour of the original melody, and to avoid tritone intervals. Altered feedback pitches occurred in one of 8 possible locations within each trial. As metrical accent strength has been found to influence both correct (error-free) music performance and the likelihood of performance errors [8,65,66] among performing musicians, half of the altered feedback locations occurred at odd-numbered serial positions in the tone sequence (aligning with strong metrical accents in the melody’s binary time signature), and the other half occurred at even-numbered serial positions (aligning with weak metrical accents in the melody’s time signature).

Examples of potential altered feedback pitches for one stimulus melody are shown in Figure 1. In the future +1 condition, participants heard the pitch that corresponded to the next intended (memorized) pitch in the melodic sequence when they pressed the piano key. In the future +2 condition, participants heard the pitch that corresponded to the intended pitch that was 2 events ahead in the melodic sequence. In the noncontextual condition, participants heard a pitch from the melody’s diatonic key (determined by the key signature of each stimulus melody) that was not present in the melodic sequence. Noncontextual pitches were chosen to match the contour and interval size as closely as possible to that of the intended pitch. The noncontextual condition was intended to serve as a control condition, to test effects of hearing an altered feedback pitch that was unrelated to performers’ planning processes. Finally, in a baseline condition, no auditory feedback pitches were altered (participants heard the intended auditory feedback). 

**Figure 2:**
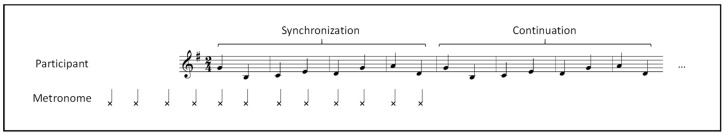
Synchronization-continuation trial. Participants synchronized the first iteration of each melody with a metronome (‘Synchronization’), and then performed two and a half additional melody iterations without the metronome (‘Continuation’). Four initial metronome beats set the performance tempo. The metronome sounded every 500 ms.

Each stimulus trial contained three and a half continuous iterations (without pausing) of a repeated melody (described below in Procedure). Each trial began with a 12-beat metronome sounded every 500 ms; the first four beats indicated the intended pace and the remaining eight beats coincided with the pianists’ first iteration of the melody, forming the synchronization phase of the trial (see Figure 2). The metronome then stopped and the pianists continued performing for two and a half more iterations of the melody, forming the continuation phase of the trial. Altered feedback pitches could occur during the continuation phase only. A minimum of zero and a maximum of two pitches were altered within a single trial, with a maximum of one altered pitch per melody iteration. When two altered pitches occurred in a single trial, they were always separated by at least three unaltered pitch events. No alterations occurred on the first pitch of any iteration or on the last four pitches of any trial. 

### 2.3. Equipment

Participants performed the stimulus melodies on a Roland RD-700SX musical instrument digital interface (MIDI) digital piano keyboard (Roland Corporation, Ontario, CA, USA) in a sound- and electrically-attenuated chamber while EEG was recorded. As pianists performed, sound was emitted from a Roland Edirol SD-50 system (Roland Corporation, Ontario, CA, USA) and delivered through EEG-compatible air-delivery earphones (ER1-14B, Etymotic Research). Two channels were used for auditory feedback: “GMT piano 002” for piano key press auditory feedback, and “Rhy 001” for the metronome that signaled the performance rate at the start of each trial. Auditory feedback pitches were controlled using FTAP version 2.1.06 [67]. FTAP presented pre-programmed pitches at the time that pianists pressed each key, and measured key press timing information with 1-ms resolution. 

### 2.4. Design

The study used a repeated measures within-participant design in which altered auditory feedback pitches were manipulated in four conditions: Future +1, future +2, noncontextual, and baseline. Participants completed trials in three blocks, each corresponding to an altered auditory feedback type (future +1, future +2, and noncontextual). Each block contained 32 trials, 50% of which contained no altered auditory feedback (baseline condition), and 50% which contained an altered feedback pitch (future +1, future +2, or noncontextual). Each trial containing altered auditory feedback was unique across the entire experiment and therefore was heard only once by participants. Block and melody orders were counterbalanced across the 24 participants. Participants performed a total of 96 (3 blocks × 32) trials, equivalent to 192 continuation iterations (32 future +1, 32 future +2, 32 noncontextual, and 96 baseline), over the course of the entire experiment. The dependent variables of the tone interonset interval (IOI), ERP component amplitudes, and theta band power were analyzed at sequential positions, −1, 0, and +1, relative to the altered tone location (as shown in Figure 1). 

### 2.5. Procedure

Participants first completed a musical background questionnaire, followed by a piano performance memory test. Participants were then presented with a short novel right-hand melody (not included in the experiment) to practice and memorize; those who were able to memorize and perform it to a note-perfect criterion within three attempts, after up to three minutes of practice with the music notation, were invited to participate in the experiment. All pianists met this criterion. Following completion of the memory test, participants were outfitted with EEG caps and electrodes. 

Participants were then asked to complete three practice trials in order to become familiar with the task. At the start of the practice trials, the participants were again presented the music notation of the single-hand melody that they had previously performed in the memory test. They were asked to indicate when they had memorized the melody. The music notation was then removed and replaced with a fixation cross. Participants were then asked to perform the melody from memory at the rate indicated by four clicks of a metronome cue (500 ms per quarter note beat). They were told that they would sometimes hear a tone that did not match the key that they pressed, but that they should keep performing at the rate cued by the metronome and try not to stop or slow down. Participants were also instructed to view the fixation cross while they were performing. The purpose of the fixation cross was to inhibit large eye movements and control participants’ gaze locations during the performance task, following other EEG studies [68,69]. During each of the three practice trials, a single feedback pitch was altered to correspond to the future +1, future +2, and noncontextual experimental conditions. The order of the three practice trials was counterbalanced across participants. 

Following the three practice trials, participants were presented with the music notation of one of the four experimental stimulus melodies. They were asked to practice the melody for a maximum of three minutes, using the notated fingering, with the goal of performing it from memory. Following memorization, the notation was removed and replaced with a fixation cross. Participants then performed the melody from memory in the synchronization-continuation trials. The first three synchronization-continuation trials contained no altered feedback, so that the experimenters could verify that participants had successfully memorized the melody; all participants were able to perform at least one of the three verification trials without producing any pitch errors. 

In each synchronization-continuation trial, participants were instructed to perform the melody from memory at the rate indicated by the metronome (500 ms per quarter-note beat), to not stop or slow down if they heard a tone that did not match the key that they pressed, and to continuously repeat the melody until they stopped hearing auditory feedback from their key presses. The metronome stopped when the participant began the second iteration of the melody. Participants were asked to refrain from moving their head or body while performing in order to minimize movement-related EEG artifacts. Eyeblinks typically create artifacts in the EEG signal, which can be addressed using a variety of artifact rejection procedures (for a review, see [70]). In order to minimize eyeblink-related artifacts, participants in some studies may be asked to refrain from blinking during certain parts of EEG trials. Since the duration of each synchronization-continuation trial in the current study exceeded 15 s, participants were not asked to refrain from blinking during the trial. Following each trial, participants indicated when they were ready to proceed to the next trial. This procedure was repeated for each of the 4 stimulus melodies and for each of the 3 feedback blocks. The synchronization-continuation trials lasted approximately 45 min. At the end of the experiment, participants were asked if they noticed any specific aspects of the altered feedback or its manipulation across the experiment; none of the participants reported an awareness of any relationship between the altered feedback and performance.

### 2.6. Data Recording and Analysis

#### 2.6.1. Behavioral Data

Behavioral disruption associated with the presentation of altered auditory feedback was evaluated by analyzing IOIs from the time of one key press to the next key press (in ms) for pitches that occurred before (position −1), during (position 0), and after (position +1) the altered auditory feedback pitch (position +1; see Figure 1). Errors in pitch accuracy were identified by computer comparison of pianists’ performances with the information in the notated musical score (Large, 1993). Pitch errors were defined as pitch additions, deletions, and corrections (errors in which pianists stopped after an error and corrected their performance). A mean of 7.9% of trials (SD = 7.3%) across subjects and conditions contained pitch errors; these trials were excluded from analyses, since any error that added or subtracted a tone from the melodic sequence changed the relationship between the participants’ key presses and the pre-programmed auditory feedback. 

#### 2.6.2. EEG Data

Electrical activity was recorded at the scalp using a 64-channel Ag/AgCl electrode BioSemi ActiveTwo System (BioSemi, Inc., Amsterdam, The Netherlands). A sampling rate of 1024 Hz, recording bandwidth of 0 to 205 Hz, and resolution of 24 bits were used. Electrode locations were prescribed by the 10–20 international electrode configuration system. Horizontal and vertical eye movements were monitored by electrodes placed adjacent to the outer canthi of the eyes and above and below the right eye, respectively. 

EEG data were analyzed using BrainVision Analyzer 2.0.2 (Brain Products GmbH, Gilching, Germany). Activity was re-referenced off-line to the average of all scalp electrodes, and signals were bandpass-filtered between 0.1 and 30 Hz. The EEG data were then segmented into 500 ms epochs beginning 100 ms prior to and continuing 400 ms after pitch onsets at positions −1, 0, and +1. Activity during the 100 ms prior to pitch onsets served as a baseline. An epoch duration of 500 ms was selected since it included activity that was shorter than three standard deviations below the mean IOI (=487 ms) of key presses recorded during the continuation period, and therefore avoided contamination of the observed waveforms with ERPs related to the subsequent pitch onset. Artifact rejection was performed automatically using a ±50 μV rejection threshold at the 64 scalp electrodes, as well as the horizontal and vertical right eye electrodes. Artifacts were considered excessive for a given subject when more than half of the epochs from a given condition of the experiment exceeded the ±50 μV rejection threshold at one of the 64 scalp electrodes or at the horizontal or vertical eye electrodes. Trials that contained pitch errors were also excluded from EEG analyses, resulting in the inclusion of 30.4/32 epochs (SD = 3.2) in the future +1 condition, 28.2/32 epochs (SD = 3.3) in the future +2 condition, 28.2/32 epochs (SD = 2.3) in the noncontextual condition, and 85.3/96 epochs (SD = 6.8) in the baseline condition (which contained three times as many stimuli as it was matched to the other conditions).

Average ERPs by participant and experimental condition were then computed for the 500-ms window time-locked to the 100 ms prior to pitch onsets. Mean ERP amplitudes were statistically evaluated at 3 topographical regions of interest (ROIs), based on related findings [7]: Anterior (electrodes Fz and FCz), central (electrodes Cz and CPz), and posterior (electrodes Pz and POz). ERP amplitudes were statistically evaluated over 40-ms time windows selected based on previous findings [7] as follows: 80–120 ms (labeled N1), 120–160 ms (labeled P2), 180–220 ms (labeled FRN), and 250–290 ms (labeled P3a). All of the ERP components were maximal at the anterior ROI; results are therefore reported for the anterior ROI only, following previous work [7,56,71]. Repeated-measures analyses of variance (ANOVAs) were conducted on ERP component amplitudes to analyze the effects of feedback type (future +1, future +2, noncontextual, and baseline). Scalp topographic maps showing ERP component distributions were generated by plotting amplitude values on the scalp. Activity was averaged across the time window used for the analysis of each component. Within-participant correlations between mean ERP amplitudes and behavioral measures for each participant were computed using simple linear regression. 

Because increases in spectral power in the theta frequency range (4–8 Hz) typically accompany the FRN [63], we analyzed theta power at the anterior ROI within the 200–300 ms that followed pitch onsets at the three event positions [7,56,72]. To allow for the specification of a temporal baseline period as well as a temporal buffer, with the purpose of preventing edge artifacts within the 100-ms epoch of interest, time-frequency decompositions were calculated for each participant in a −1000 to +1000 ms time window centered on pitch onsets [73]. Our goal in using time-frequency analysis was to ensure that any potential ERP component overlap in the average ERP waveforms did not provide an alternative interpretation of our results. In order to eliminate influences of faster or slower components overlapping the FRN in the average ERP waveforms, decompositions were computed using a Morlet wavelet transform based on each participant’s average ERP waveforms for each experimental condition [56,64,74]. To achieve sufficient temporal resolution for the theta frequency range, the number of Morlet wavelet cycles used for analysis of the theta band was set to *n* = 7 [75,76]. Mean power in a pre-stimulus baseline period of −100 to 0 ms was subtracted from the 2-s time-frequency analysis window to permit the assessment of event-related changes in theta activity [77]. Repeated-measures ANOVAs on mean theta power within the 200–300 ms following pitch onsets with factors’ feedback type (future +1, future +2, noncontextual, baseline) and event position (0, +1) were conducted to analyze the effects of feedback conditions on theta power. Post-hoc pairwise comparisons were made using Tukey’s honestly significant difference (HSD) test for both behavioral and neural measures. $\eta_{p}^{2}$ was used as a measure of effect size.

## 3. Results

### 3.1. Future +1 Altered Feedback Disrupts Key Press Timing

The mean performance rate, indicated by the mean IOI per trial, during the continuation phase of the synchronization-continuation trials was 486.5 ms (SE = 0.3 ms), slightly faster than the metronome-indicated rate of 500 ms from the earlier synchronization phase. An ANOVA on mean IOIs per trial within the continuation phase by feedback condition yielded no main effect of feedback, F (3, 69) = 1.78, *p* = 0.16, suggesting that performance rates did not differ across the four conditions (future +1 *M* = 485.8 ms, SE = 0.5; future +2 *M* = 486.2 ms, SE = 0.6; noncontextual *M* = 486.4, SE = 0.5; baseline *M* = 487.7, SE = 0.5). Thus, performers successfully maintained the same tempo for all feedback conditions, with slightly faster rates than the prescribed rate overall, consistent with similar previous studies [7,78]. 

**Figure 3:**
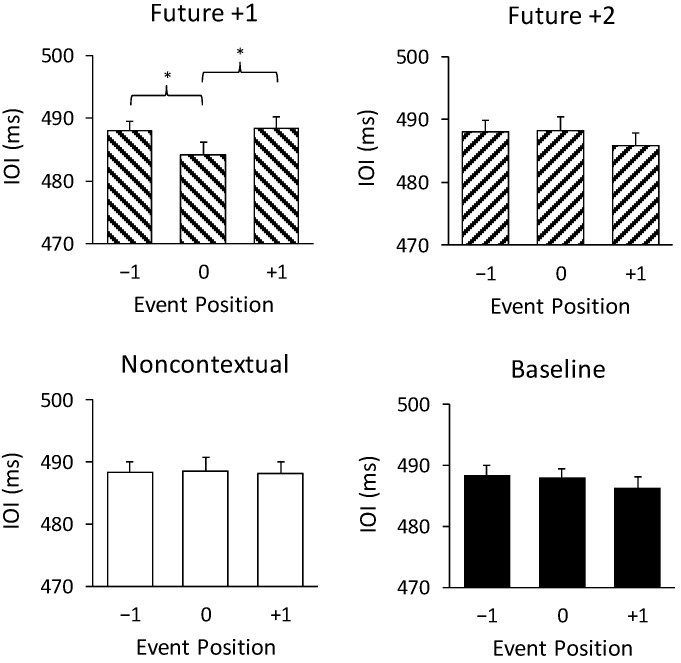
Pianists’ mean interonset intervals (IOIs) by altered feedback condition (baseline, future +1, future +2, and noncontextual) by target event position (−1, 0, +1). Error bars represent one standard error. * *p* < 0.05.

Figure 3 shows IOIs at melody positions preceding, at, and following at the altered feedback pitches and the same positions in the unchanged baseline pitches. An ANOVA on mean IOIs by feedback condition (future +1, future +2, noncontextual, baseline) and event position (−1, 0, +1) revealed a significant interaction of feedback condition with event position, F (6, 138) = 3.60, *p* < 0.005, $\eta_{p}^{2}$ = 0.14. IOIs at position 0 were significantly shorter than IOIs at positions −1 and +1 for the future +1 feedback condition only (Tukey HSD = 3.81, *p* < 0.05). IOIs did not significantly differ between positions −1, 0, and +1 for any other condition. There were no main effects of feedback type or position on IOIs. Thus, the only condition in which the altered auditory feedback temporally disrupted performance was the future +1 feedback condition, in which performers shortened the time interval during which they heard the altered feedback tone.

We next analyzed participants’ key press errors (7.9% of all trials) by feedback condition. There was no significant main effect of feedback condition on the mean proportion of trials that contained errors, F (3, 69) = 0.37, *p* = 0.78. Pitch errors occurred at roughly equivalent rates across trials in all four feedback conditions (future +1 *M* = 7.8%, SE = 1.7%; future +2 *M* = 9.0%, SE = 1.5%; noncontextual *M* = 7.5%, SE = 1.2%; baseline *M* = 7.1%, SE = 1.6%). 

### 3.2. EEG Results

#### 3.2.1. Event-Related Potentials

**Figure 4:**
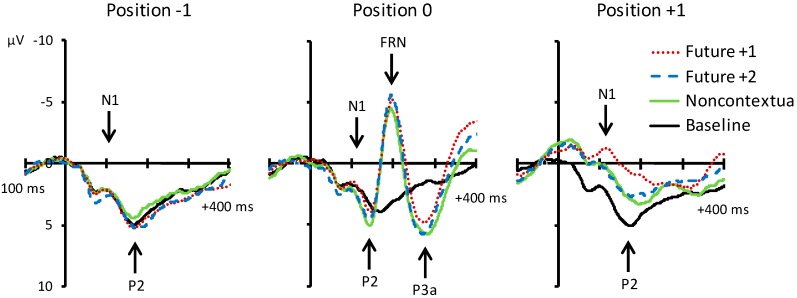
Grand average event-related potentials (ERPs) elicited by the four experimental conditions relative to target event positions −1, 0, and +1. Activity shown is averaged across all electrodes contained within the anterior region of interest (ROI). Negative is plotted upward.

**Figure 5:**
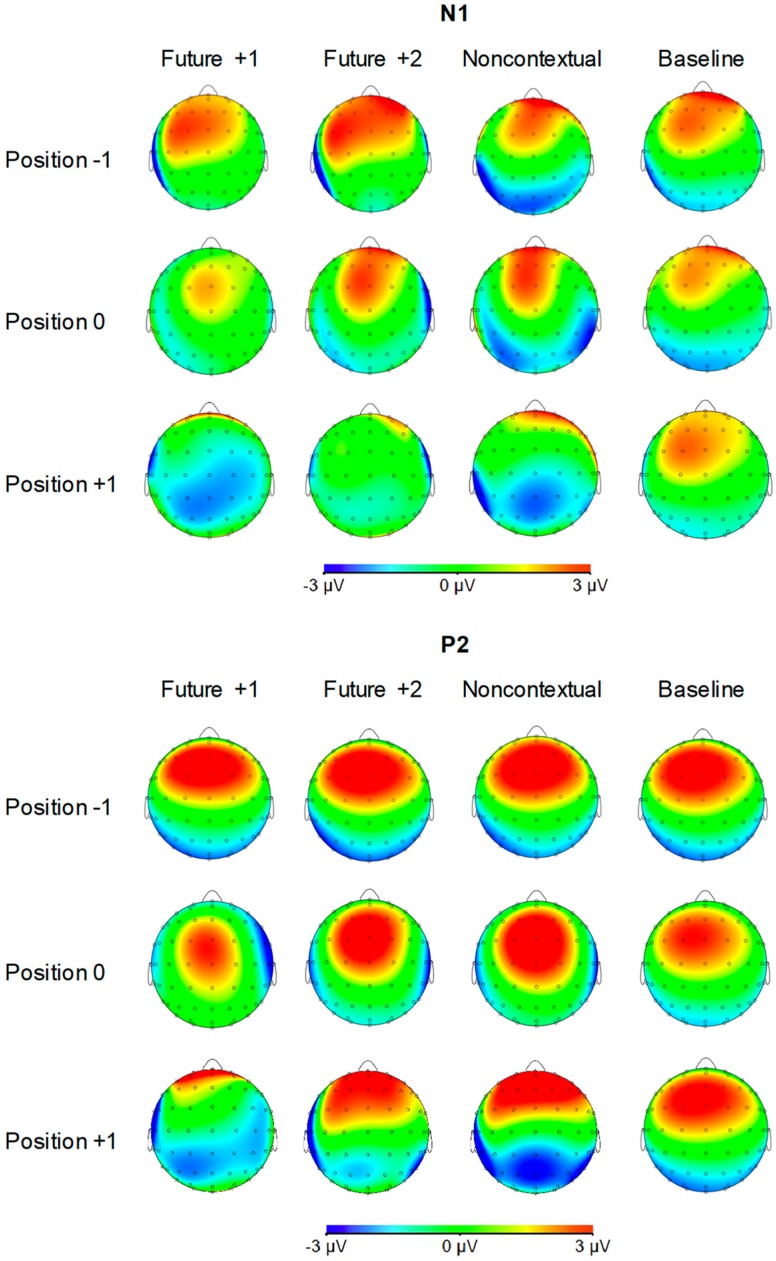
Voltage (in µV) scalp topographies of N1 and P2 components relative to target event positions −1, 0, and +1 by feedback condition. Activity averaged over 40 ms surrounding each component’s grand average peak is shown.

**Figure 6:**
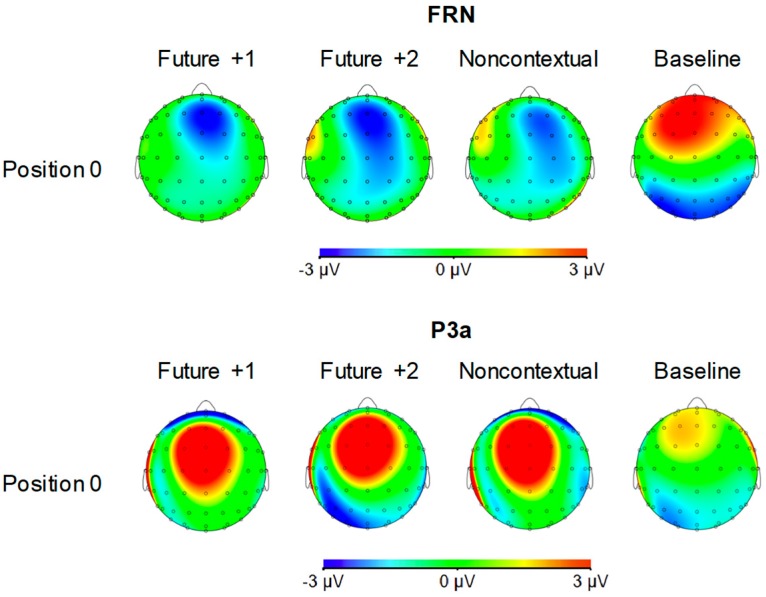
Voltage (in µV) scalp topographies of feedback-related negativity (FRN) and P3a components elicited by pitches at target event position 0 by feedback condition. Activity averaged over 40 ms surrounding each component’s grand average peak is shown.

Figure 4 shows grand averaged ERP waveforms time-locked to key press onsets, averaged across error-free trials. ERP components are time-locked to key presses corresponding to the feedback pitch onset at position 0, as well as to the key presses at melody positions −1 (preceding location) and +1 (following location). N1 components and P2 ERP components, labeled in Figure 4, were observed at positions −1, 0, and +1 for all feedback conditions. Additionally, FRN and P3a components were observed at position 0 for the three altered feedback conditions. Scalp topographies corresponding to the N1 and P2 components at positions −1, 0, and +1 by feedback condition are shown in Figure 5. Topographies corresponding to the FRN and P3a components at position 0 are shown in Figure 6. Analyses of each ERP component are reported in turn.

*N1 component (80–120 ms).* We first evaluated whether mean amplitudes within the N1 time window differed across auditory feedback conditions. We conducted one-way ANOVAs on N1 amplitudes at each event position with the factor feedback type. N1 amplitudes did not significantly differ across feedback conditions at position −1, F (3, 69) = 0.18, *p* = 0.91. N1 amplitudes also did not significantly differ across feedback conditions at position 0, F (3, 69) = 1.47, *p* = 0.23. Analysis of N1 amplitudes at position +1 yielded a significant main effect of feedback type, F (3, 69) = 7.42, *p* < 0.001, $\eta_{p}^{2}$ = 0.24. All three altered feedback types elicited a significantly more negative N1 than did baseline feedback pitches (Tukey HSD = 1.73, *p* < 0.05). Thus, N1 amplitudes at event position +1 were sensitive to whether altered auditory feedback was presented one tone earlier (altered feedback conditions) or not (baseline condition). Specifically, N1 amplitudes were more negative following altered compared to baseline feedback. 

*P2 component (120–160 ms).* We next evaluated whether mean amplitudes within the P2 time window differed across auditory feedback conditions. We conducted one-way ANOVAs on P2 amplitudes at each event position with the factor feedback type. P2 amplitudes did not significantly differ across feedback conditions at position −1, F (3, 69) = 0.25, *p* = 0.86. P2 amplitudes also did not significantly differ across feedback conditions at position 0, F (3, 69) = 1.04, *p* = 0.38. Analysis of P2 amplitudes at position +1 yielded a significant main effect of feedback type, F (3, 69) = 13.95, *p* < 0.001, $\eta_{p}^{2}$ = 0.38. All three altered feedback types elicited a significantly less positive P2 than baseline feedback pitches (Tukey HSD = 2.12, *p* < 0.01). Furthermore, the P2 elicited by future +1 feedback was significantly less positive than the P2 elicited by future +2 and noncontextual altered feedback (Tukey HSD = 1.73, *p* < 0.05). Thus, like the N1 component, the P2 was sensitive to whether altered auditory feedback was presented one tone earlier or not. Critically, P2 amplitudes were more negative following future +1 feedback compared to future +2 feedback. 

*Correlation of N1 and P2 amplitudes.* The temporal proximity of N1 and P2 components as well as their co-occurrence following both altered and unaltered feedback is consistent with their interpretation as joint indices of auditory sensory processing [34]. To test the relationship between N1 and P2 components, mean amplitudes within the N1 time window (80–120 ms) were compared with amplitudes within the adjacent P2 time window (120–160 ms) for each position and feedback condition. As shown in Table 1, amplitudes within the time windows of the N1 and P2 were significantly correlated for all feedback conditions at positions −1, 0, and +1 (all *p*s < 0.001). 

*FRN component (180–220 ms).* Analysis of mean amplitudes within the FRN time window at position 0 yielded a significant main effect of feedback type, F (3, 69) = 31.53, *p* < 0.001, $\eta_{p}^{2}$ = 0.58. All three altered feedback types elicited a significantly more negative FRN compared to the baseline condition (Tukey HSD = 2.58, *p* < 0.05). No other comparisons reached significance. Thus, all three altered auditory feedback types elicited an FRN response. 

*P3a component (250–290 ms).* Analysis of mean amplitudes within the P3a time window at position 0 yielded a significant main effect of feedback type, F (3, 69) = 7.70, *p* < 0.001, $\eta_{p}^{2}$ = 0.25. All three altered feedback types elicited a significantly more positive P3a compared to the baseline condition (Tukey HSD = 2.44, *p* < 0.05). No other comparisons reached significance. Thus, as predicted, all three altered auditory feedback types elicited a P3a response.

#### 3.2.2. Evoked Oscillatory Responses

**Figure 7:**
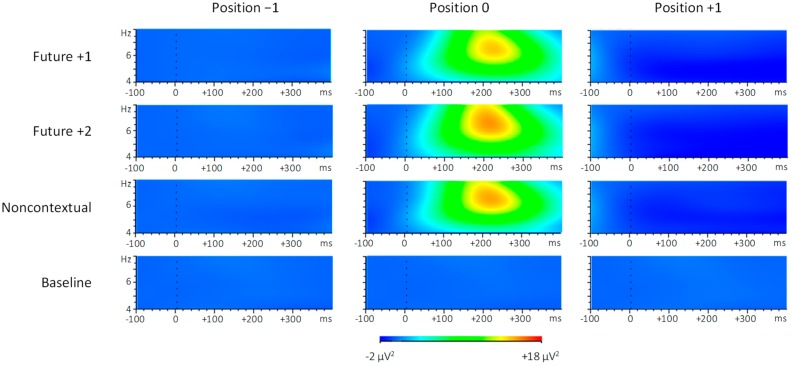
Evoked spectral power within the 4-8 Hz (theta) frequency range following pitch onsets at target event positions −1, 0, and +1. Brighter colors indicate greater spectral power.

To assess whether altered auditory feedback influenced spectral power within the theta frequency range, we computed spectral power in the 4–8 Hz frequency range within the anterior ROI at each event position, as shown in Figure 7. Analysis of theta spectral power during the 200–300 ms following pitch onsets by feedback condition (future +1, future +2, noncontextual, and baseline) and position (−1, 0, and +1) yielded main effects of both feedback condition, F (3, 69) = 6.49, *p* = 0.001, $\eta_{p}^{2}$ = 0.22, and position, F (2, 46) = 8.24, *p* = 0.001, $\eta_{p}^{2}$ = 0.26. There was also a significant interaction between feedback condition and position, F (6, 138) = 7.68, *p* < 0.001, $\eta_{p}^{2}$ = 0.25. Theta power was greater for each of the three altered feedback conditions compared to the baseline feedback condition at position 0 (Tukey HSD = 157.2, *p* < 0.01). Theta power was also greater at position 0 compared to position +1 within each of the three altered feedback conditions (Tukey HSD = 157.2, *p* < 0.01). In sum, theta power increased only following altered feedback pitches that occurred at position 0, and not following (unaltered) feedback pitches that occurred at position +1. Thus, changes in theta power depended on whether the feedback was altered or not, and not on whether the feedback contents were repeated (future +1) or not (future +2). 

### 3.3. Correlations of Neural and Behavioral Measures

#### ERP Amplitudes and IOIs

**Figure 8:**
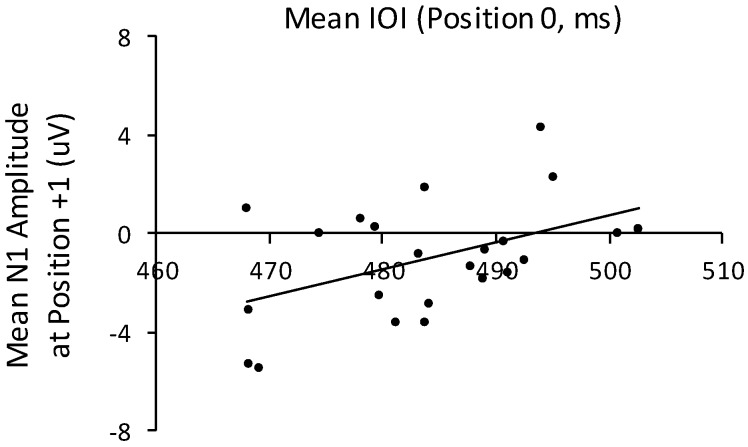
Correlation of mean IOIs at target event position 0 in the future +1 altered feedback condition with mean N1 amplitudes elicited by the tone that followed the altered auditory feedback pitch in the future +1 condition. Each dot represents one participant.

To examine the relationship between the temporal disruption to key press timing and the ERP components, we first tested whether the temporal disruption arising from future +1 auditory feedback—the shortening of the position 0 IOI—correlated with mean amplitudes of ERP components at position +1 that immediately followed the disrupted timing. As shown in Figure 8, the shortened mean IOIs at position 0 correlated significantly with mean amplitudes of the subsequent N1 in the future +1 condition, *r* (22) = 0.47, *p* < 0.05. Shorter IOIs at position 0 were associated with a larger N1 response to the pitch that followed the altered feedback. The correlation of mean IOIs at position 0 with amplitudes of the P2 at position +1 yielded a similar pattern of association, but the correlation did not reach significance, *r* (22) = 0.27, *p* = 0.22. Mean N1 and P2 amplitudes did not correlate with mean IOIs at position 0 for any other feedback condition (future +2, noncontextual, and baseline feedback). Thus, auditory sensory processing of the tone following the altered feedback, reflected in the N1, was associated with temporal disruption only when near future altered feedback was presented.

**Figure 9:**
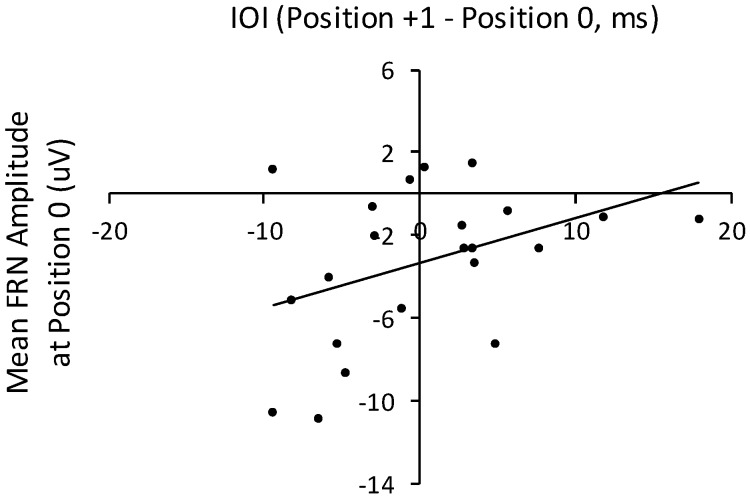
Correlation of mean IOI differences (target event position 1 minus position 0) from the three altered feedback conditions (future +1, future +2, and noncontextual) with mean FRN amplitudes elicited by altered feedback (target event position 0) across the three altered feedback conditions (future +1, future +2, and noncontextual). Each dot represents one participant.

To examine the relation between temporal disruption and FRN responses to altered auditory feedback, we computed the interonset change (in ms) from the IOI at position 0 to the IOI at position +1. Participants’ mean difference in IOIs between positions 0 and +1 across all three altered feedback conditions correlated significantly with mean FRN amplitudes time-locked to the altered feedback pitch (position 0), *r* (21) = 0.41, *p* < 0.05, shown in Figure 9. Mean amplitudes within the time window of the FRN were not correlated with the difference in IOIs across positions 0 and +1 for the baseline condition, *r* (21) = 0.27, *p* = 0.24. Therefore, amplitudes of the FRN elicited by altered auditory feedback were associated with changes in the performance rate that succeeded the altered feedback: More negative FRNs were associated with increases in the performance rate. No other ERP component amplitudes correlated significantly with IOIs or with IOI differences at event positions preceding or following altered auditory feedback.

## 4. Discussion

We examined the relationship between future-oriented planning processes and feedback monitoring during music performance. Skilled pianists performed short melodies from memory. Perceived auditory feedback was occasionally altered to match immediately upcoming sequence events (future +1), later future events (future +2), or unrelated pitches that were not contained within the performed sequences (noncontextual). There were several novel findings. First, only future +1 altered feedback—not future +2 or noncontextual altered feedback—perturbed the timing of pianists’ key presses. Second, the length of time it took performers to initiate the pitch following the future +1 altered feedback pitch was associated with larger auditory sensory potentials to the post-altered feedback pitch. Third, all types of altered feedback elicited FRN and P3a potentials. Fourth, FRN amplitudes increased as performers sped up following the altered feedback pitch, in response to all types of altered auditory feedback. Together, these findings suggest that future-oriented planning during production influences how performers monitor their auditory feedback. The range of sequential planning may be constrained by distance: Events at nearby sequence positions had a greater influence on planning and monitoring processes than did events at farther positions, consistent with theories of sequence production in which planned events are activated along a gradient that is defined by sequential distance [8,9,12]. According to a predictive coding model [15], a cascade of forward models for upcoming movements may generate an error signal in response to altered auditory feedback that is stronger when the feedback matches nearby sequence positions than when it matches farther positions.

### 4.1. Behavioral Findings

The timing of pianists’ performances was disrupted following the perception of altered auditory feedback that corresponded to near future, but not far future, events. According to future-oriented theories of planning during music and speech production, immediately upcoming events receive stronger activation than events that are farther ahead in a melody or utterance [8,12]. When pianists heard an altered feedback pitch that matched an event that was already strongly activated in memory, the altered pitch may have generated similarity-based interference with the event that was currently being produced. Thus, temporal perturbations observed in the future +1 condition may reflect the greater interference of near future altered feedback with currently planned pitch events compared to far future altered feedback. This interpretation is consistent with theories of sensorimotor production in which actions and their auditory effects share common cognitive representations [79,80], as well as theories in which actions are planned in terms of their sensory effects [81,82,83]. We previously demonstrated that future-oriented, but not past-oriented, altered auditory feedback induced compensatory adjustments in keystroke timing [7]. The current results extend this finding by suggesting that future-oriented interference interacts with graded planning and monitoring processes during music performance. 

Another important factor that constrains memory retrieval of sequence of events is the similarity between sequence elements. Evidence from production errors and priming paradigms has indicated that grammatical and phonological similarity influence lexical retrieval [84,85], and tonal and metrical accent relationships influence event retrieval during music performance [8,65]. For example, musicians are more likely to produce pitch errors in metrically weak than in metrically strong accent positions [65]; sequence events that align with greater metrical accent strength tend to be produced with greater intensity [86]. The melodies used in the current study were designed so that metrical accents of the future +2 altered feedback pitch were more similar to the currently planned pitch event than were the metrical accents of future +1 feedback [87]. This metrical similarity approach would predict that the future +2 altered feedback should generate greater interference and performance disruption than future +1 feedback. This prediction was not supported by the current results: Instead, altered feedback that contained serially proximal pitches was more disruptive to performance than altered feedback that contained metrically similar pitches. This suggests that serial proximity may play a greater role than metrical accent strength in generating interference with planned representations for the short sequences used in the current study. One explanation for the lesser contribution of metrical accent to the disruptive effects of altered auditory feedback could be that metrical relationships between sequence events tend to span longer timeframes than the timespans between serially proximal events [65]. 

Serially-shifted feedback, like the future-oriented altered auditory feedback presented in our study, is known to increase performers’ overall key press error rates [21]. We observed heightened error rates in all altered auditory feedback conditions compared to baseline (unchanged) feedback. Error rates were relatively low compared to rates as high as 40% observed in other studies employing serially-shifted auditory feedback [22]. A likely explanation for this difference is that single pitches were altered at random sequence locations in the current study, which prevented performers from anticipating the alterations, unlike in previous studies, in which auditory feedback was continuously and consistently altered. When auditory feedback is predictably altered, performers can develop strategies to compensate for predictable deviations from expected feedback. Even under conditions in which every feedback tone is altered during music performance, pitch errors begin to occur only after several melody repetitions [88]. Future studies could further investigate interactions between hierarchical and distance constraints on sequence planning using musical materials that amplify differences between strongly and weakly accented events.

### 4.2. EEG Findings

Altered auditory feedback attenuated cortical sensory suppression compared to baseline feedback, reflected in amplitude-shifted N1 and P2 ERP components. Sensory suppression is widely believed to result from the congruence between sensory consequences of actions and sensory predictions generated by forward models of motor commands (for a review, see [29]). Theories of motor control have proposed that efference copies of motor commands are used to predict sensory outcomes of those commands, and that sensory suppression results from the subtraction of an efference copy from actual sensory input [14,89]. Sensory suppression is often used as an implicit measure of agency, as actions must be volitional in order to generate predictive models of motor commands [90,91]. Increased auditory sensory processing following altered feedback pitches could therefore indicate that altered feedback disrupted pianists’ sense of agency or control over the sounds that they were producing (cf. [7]). This interpretation also fits with the proposal that sensory suppression during production may serve the purpose of allowing producers to differentiate self-generated from externally-generated sensations [36]. 

We observed a greater reduction of sensory suppression following future +1 altered feedback compared to future +2 altered feedback, reflected in the P2. This finding suggests that the post-altered feedback pitch received enhanced cortical sensory processing in the future +1 condition compared to the future +2 condition. Further, reduced sensory suppression in the future +1 condition was associated with a quicker initiation of the tone following the future +1 feedback pitch. Together, these findings suggest that enhanced cortical sensory processing following the future +1 altered auditory feedback may have aided the recovery from perturbations caused by the unexpected feedback. Indeed, expectancy violations tend to receive enhanced neural processing compared to events that fulfill expectations, in line with a predictive coding view of cortical responses [92]. It is unlikely that differences in P2 amplitudes for future +1 and future +2 conditions were driven by differences in selective attention between altered feedback conditions, since sensory suppression during auditory production appears to be uninfluenced by whether attention is directed toward or away from one’s own actions or their auditory effects [93]. It is also unlikely that this amplitude difference is due to differences between future +1 and future +2 conditions in terms of pitch repetition. From a repetition suppression perspective, we would expect decreased—not increased—cortical processing of the tone that followed the future +1 altered feedback tone, since this tone was repeated and stimulus repetition classically results in a decreased brain response due to sensory adaptation [94,95]. The fact that theta power did not distinguish future +1 from future +2 responses supports this interpretation. We propose that sensory suppression depended on the differences in interference generated by the future +1 and future +2 altered feedback pitches with concurrent planning processes. Amplitudes may indicate the degree of conflict or mismatch between perceived altered auditory feedback and concurrent planning processes, which are biased towards the immediate future. 

Both N1 and P2 components are sensitive to a variety of acoustic features of incoming auditory signals, highlighting a role of these components in early auditory sensory processing. For example, pitch changes in vocal stimuli during active vocalization elicit larger N1 and P2 responses than pitch changes in non-voice complex stimuli, which in turn elicit larger amplitudes than pure tones [96]. Acoustic spectral complexity [42], pitch discrimination and speech-sound training [43,97], and the rate of speech formant transition [98] have all been shown to modulate N1 and P2 responses. The current results extend these findings by demonstrating that N1 and P2 amplitudes also take into account the relationship between pitch changes and planned events in an auditory sequence. Speech sounds are generally more spectrally complex than musical sounds [99]. An open question for future research is therefore whether alterations of auditory feedback during speech production are better detected by the auditory system than feedback alterations during music performance.

FRN and P3a ERP components were elicited by all altered auditory feedback (future +1, future +2, and noncontextual) pitches. ERP amplitudes were equivalent across all altered feedback conditions. FRN and P3a components have been elicited by altered auditory feedback during music performance in previous studies [45,46,47]. None of these studies compared neural responses to different types of altered auditory feedback, with the exception of Katahira and colleagues [45], who manipulated the diatonicity of altered feedback tones. The current finding suggests that performers identified and subsequently oriented toward all types of unexpected feedback. This finding fits with the principle that any alteration of feedback during auditory-motor tasks creates a mismatch between movements and expected auditory outcomes, which create larger violations for producers with higher skill levels [19] or with greater sequence familiarity [100]. Studies using flanker gambling tasks have demonstrated that the FRN is sensitive to the perceptual distinctiveness of unexpected stimuli [101,102,103]. The noncontextual control condition presented diatonically-related altered feedback pitches that were more distinct from the pitch set of the produced melodies than were the altered pitches in the future +1 and future +2 melodies. Yet, the FRN elicited by noncontextual altered feedback did not differ from that elicited by contextual (future +1 and future +2) feedback. The association between FRN amplitudes and speed of the altered feedback pitch for all three altered feedback conditions further supports this interpretation. Thus, FRN responses may be less affected by perceptual distinctiveness or by performers’ planning processes and more dependent on action-related expectations. Future studies may address this possibility directly with manipulations of perceptual distinctiveness.

Theta power increases were also observed following all types of altered feedback tones, about 200–300 ms after the altered pitch onsets. The lack of differences in theta power across feedback conditions confirms the FRN results, and suggests that amplitudes of the FRN elicited by altered feedback were unaltered by overlapping ERP components. Increases in theta power within the approximate timeframe of the FRN component suggest that identification of expectancy violating pitches coincided with the emergence of a more cognitively controlled, deliberative mental state, as opposed to a mental state relying primarily on habit or performance routines [63]. Just as the FRN has been suggested to reflect surprising action-based outcomes [104], theta has been referred to as a “surprise signal” that leads to task-specific adjustments in cognitive control [63]. Theta frequency oscillations may coordinate the excitability of populations of mid-frontal neurons, thereby providing a temporal window in which cognitive control can be instantiated [105]. Orienting to altered auditory feedback during music performance may therefore involve a switch from a state of relatively automatic performance to performance that is more deliberative and goal-directed, characterized by transitory changes in the production rate. Finally, increases in theta power did not differentiate between altered feedback types. Similar to the notion that the FRN may depend more on action-related expectations than on performers’ planning processes, equivalent increases in theta across feedback conditions suggest that any violation of any action-sound association is sufficient for invoking the need for cognitive control.

## 5. Conclusions

This study provides the first neural support for the finding in speech production and music performance that planning of upcoming events in a sequence is influenced by the serial proximity of the future events. Feedback monitoring processes interacted with planning processes: Performers’ perception of altered feedback tones that matched immediately upcoming future events resulted in behavioral and neural adaptations, including temporal disruption (speeded IOIs), enhanced cortical sensory processing following the altered feedback (amplitude-shifted N1 and P2 responses), and increased theta frequency activity. These findings support models of sequence production in which the planning of future events is modulated by their serial distance from the current event [8,12], and contribute to our understanding of the link between sensory suppression and action planning during the performance of complex action sequences. The N1-P2 complex may serve as a neural marker for disruptive effects of altered auditory feedback in sensorimotor tasks. 

## Figures and Tables

**Table 1:** Correlations of mean N1 and P2 amplitudes at target event positions −1, 0, and +1 for each feedback condition. * *df* = 22, *p* < 0.001.

	Position −1	Position 0	Position +1
Future +1	0.84 *	0.90 *	0.79 *
Future +2	0.94 *	0.64 *	0.82 *
Noncontextual	0.81 *	0.96 *	0.96 *
Baseline	0.64 *	0.73 *	0.82 *
